# Development of the Hypothalamic Melanocortin System

**DOI:** 10.3389/fendo.2013.00038

**Published:** 2013-03-27

**Authors:** Berengere Coupe, Sebastien G. Bouret

**Affiliations:** ^1^Neuroscience Program, The Saban Research Institute, Children’s Hospital Los Angeles, University of Southern CaliforniaLos Angeles, CA, USA; ^2^U837, Neurobese Lab, INSERM, Jean-Pierre Aubert Research Center, University Lille 2Lille, France

**Keywords:** pro-opiomelanocortin, αMSH, MC4R, hypothalamus, development, hormones, axon guidance, neurogenesis

## Abstract

The melanocortin system is a critical component of the forebrain and hindbrain regulatory systems involved in energy balance. This system is composed of pro-opiomelanocortin (POMC) neurons that act, in part, through the melanocortin-4 receptor (MC4R). Although the importance of the melanocortin system in controlling feeding has been established for two decades, the understanding of the developmental substrates underlying POMC and MC4R neuron development and function has just begun to emerge. The formation of the melanocortin system involves several discrete developmental steps that include the birth and fate specification of POMC- and MC4R-containing neurons and the extension and guidance of POMC axons to their MC4R-expressing target nuclei. Each of these developmental processes appears to require specific sets of genes and developmental cues that include perinatal hormones. Recent evidence has also highlighted the importance of perinatal nutrition in controlling the ultimate architecture of the melanocortin system.

## Introduction

**Figure 1:**
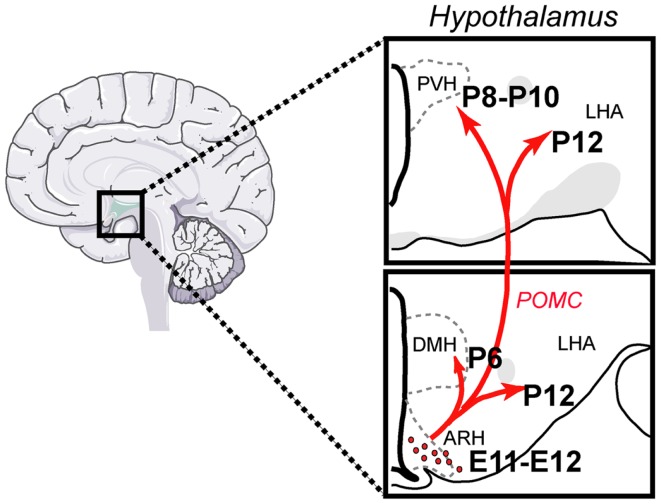
**Architecture and development of the POMC neural system in the mouse**. POMC neurons located in the arcuate nucleus of the hypothalamus (ARH) send direct projections to discrete populations of neurons located in the dorsomedial (DMH) and paraventricular nuclei (PVH) of the hypothalamus and to the lateral hypothalamic area (LHA). Each of these regions plays a major role in the control of energy balance and feeding behavior. ARH POMC neurons are born at E11–E12 and innervate their target nuclei primarily during the first 2 weeks of postnatal life.

Pro-opiomelanocortin (POMC) neurons control a variety of physiological functions, the most characterized of which is the regulation of energy balance. They reduce food intake and increase energy expenditure by releasing α-melanocyte-stimulating hormone (αMSH), a product of POMC processing (Cone, 2006; Ellacott and Cone, 2006). More recent targeted deletion studies have specifically shown the importance of POMC neurons in mediating the physiological actions of metabolic hormones, such as leptin and insulin (Belgardt and Brüning, 2010; Williams and Elmquist, 2012). POMC neurons have a limited distribution across the central nervous system (CNS). POMC cell bodies are found only in two CNS nuclei: the arcuate nucleus of the hypothalamus (ARH) in the forebrain and the nucleus of the tractus solitarius (NTS) in the brain stem (Joseph et al., 1983; Cone, 2005). Recent data indicated that while hypothalamic POMC neurons appear to be more involved in integrating long-term adiposity signals, the contribution of hindbrain POMC neurons seem to be more specific to the integration of short-term satiety signals (Zhan et al., 2013). POMC neurons provide extensive projections to various parts of the brain, especially to the paraventricular (PVH) and dorsomedial (DMH) nuclei of the hypothalamus, the lateral hypothalamic area (LHA), and the ventral tegmental area (VTA). Each of these nuclei and areas also plays a major role in the central regulation of feeding behavior and energy balance (King and Hentges, 2011) (Figure 1).

Of the five known melanocortin receptors (MCRs), αMSH binds with various affinities to MC1R, MC3R, MC4R, and MC5R. However, it is the MC4R subtype that has been primarily implicated in the regulation of energy balance. The observation that deletion of the MC4R results in a phenotype that is a virtual carbon copy of POMC-deficient mice (including late onset of obesity and diabetes) strongly supports this idea (Huszar et al., 1997; Yaswen et al., 1999; Butler et al., 2001; Challis et al., 2004). Notably, mutations in the MC4R gene are the most common monogenic disorders that cause obesity in humans (Coll et al., 2007). MC4R is a G-protein-coupled receptor that has a widespread distribution throughout the CNS. MC4R mRNA is found in virtually every part of the brain, including the hypothalamus, thalamus, cortex, brainstem, and spinal cord (Mountjoy et al., 1994; Kishi et al., 2003). Soon after the cloning of the MC4R, agouti-related protein (AgRP), which is synthesized in the ARH, has been identified as an endogenous antagonist of the MC4R (Huszar et al., 1997; Ollmann et al., 1997).

This review attempts to summarize our current understanding of the development of the melanocortin system within the context of metabolic programing. Because of the lack of data on the development of POMC neurons in the NTS, this review will largely focus on the development of arcuate POMC neurons and their related hypothalamic MC4R pathways.

## Development of the Melanocortin System

The developmental processes that produce the melanocortin system can fall into two broad categories: the birth and determination of the neuronal phenotype of POMC- and MC4R-containing neurons and the formation of functional circuits, which includes POMC axon growth and synaptogenesis onto MC4R-responsive targets.

### Neurogenesis

**Figure 2:**
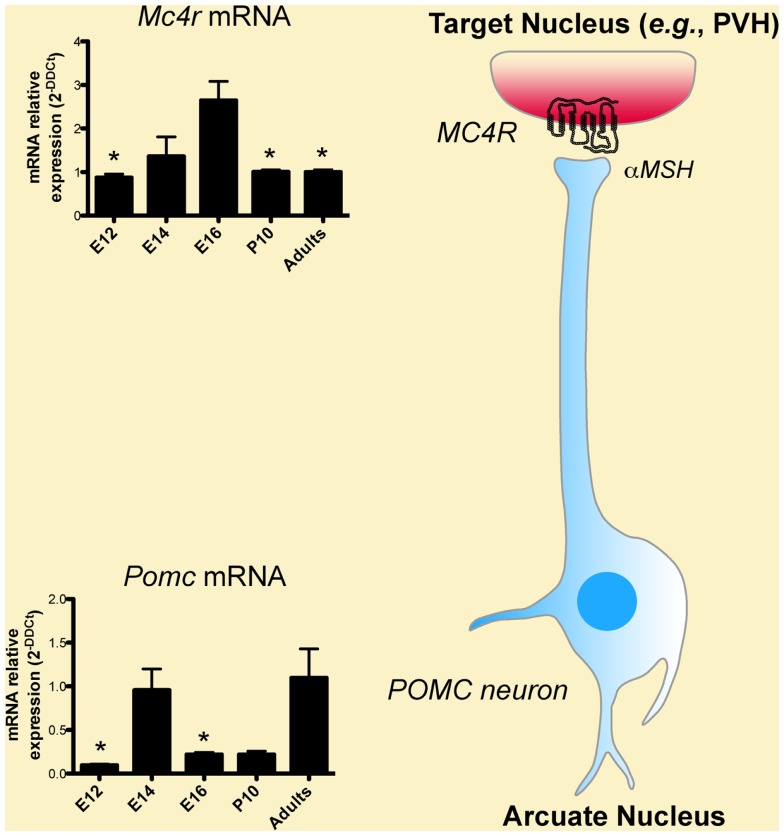
**Developmental regulation of the melanocortin system**. Low levels of *Pomc* mRNA are found in the hypothalamus as early as embryonic day (E)12, i.e., soon after the birth of POMC neurons. *Pomc* mRNA exhibits a peak of expression on E14. POMC neurons act in part through the melanocortin-4 receptor (MC4R) to exert their effects. *Mc4r* mRNA is also found in the hypothalamus as early as on E12 and display a peak of expression on E16.

Classic experiments that used birth dating tools, such as the thymidine analog bromodeoxyuridine, revealed that the majority of POMC neurons in the mouse ARH are born primarily on embryonic day (E)11–E12 (Khachaturian et al., 1985; Padilla et al., 2010). However, some POMC neurons, which are located more laterally in the ARH, are generated as late as E13. Gene expression studies have also shown that neurons in the presumptive ARH first express POMC mRNA on E10–E12 (Padilla et al., 2010) (Figure 2), which is consistent with early determination of cell fate. Recent genetic cell lineage tracing studies confirmed this hypothesis and revealed that POMC neurons acquire their terminal phenotype at approximately E15 (Padilla et al., 2010). However, only a portion of embryonic *Pomc*-expressing precursors adopts a POMC fate in adult mice. Half of *Pomc*-expressing precursors acquire a non-POMC fate in adult mice and nearly one quarter of the mature NPY neurons in the ARH share a common progenitor with POMC cells (Padilla et al., 2010). These data show the unique property of *Pomc*-expressing progenitors with respect to giving rise to antagonistic neuronal populations.

### Development of POMC-containing axonal projection

The ontogeny of the POMC neuronal projections remains to be determined specifically. However, axonal tract tracing experiments in mice have revealed that ARH projections are largely immature at birth and develop postnatally during the first 2 weeks of postnatal life. By P6, ARH projections extend through the periventricular zone of the hypothalamus to provide inputs to the DMH first, followed by inputs to the PVH between P8 and P10. Projections from the ARH to the LHA develop significantly later, with the mature pattern of innervation first apparent on P12. Not until P18 does the pattern of ARH axonal projections achieve a distribution that resembles that seen in the adult (Bouret et al., 2004a). These findings suggest that the development of ARH POMC axonal projections toward each of their target nuclei does not occur until the second week of postnatal life, i.e., at a time that is far removed from the birth of these neurons (E11–E12). At the synaptic levels, Colmers and collaborators showed that there is an age-dependent increase in the electrophysiological response of specific sets of PVH neurons to melanocortin, with a maximal response observed at P28–P35 (Melnick et al., 2007). These results suggest that synapses between POMC axons and PVH MC4R-containing neurons are not structurally and functionally mature until puberty.

### Developmental regulation of MC4R

In spite of the importance of MC4R in energy balance regulation, we still know relatively little about the exact time point at which the assembly of this system is fully established. For example, we still do not know when MC4R neurons are born or when MC4R becomes functional and able to signal. Nevertheless, the fact that peripheral injection of the MCR agonist MTII reduces milk intake and body weight as early as during the first 2 weeks of postnatal life suggests that MC4R receptors might be present and functional in the hypothalamus at this age (Glavas et al., 2007). Consistent with this idea, *in situ* hybridization analysis shows that MC4R mRNA is abundant in the hypothalamus and especially in the PVH at P10 (Figure 2). That peripheral injection of MTII induces strong activation of cFos immunoreactivity (a marker of neuronal activation) in the PVH at P5–P15 further supports the functionality of MC4R in the PVH during early postnatal life (Glavas et al., 2007). At the molecular level, it was shown that MC4R mRNA is first expressed at E12 in the proliferative zone surrounding the lower portion of the third ventricle (also known as the neurepithelium) and that this mRNA expression peaks at E16 (Figure 2) (Mountjoy and Wild, 1998). These findings are particularly interesting because it is known that neurons that compose various hypothalamic nuclei in adults are primarily derived from precursors that originate from this proliferative zone, raising the possibility that MC4R could be involved in hypothalamic neurogenesis. Supporting this hypothesis, melanocortins stimulate astrocyte proliferation *in vitro* (Zohar and Salomon, 1992). Other brains sites express MC4R during embryonic development. In addition to being expressed in the diencephalic neurepithelium, MC4R mRNA is also found in the telencephalon and the lamina terminalis at E14 (Mountjoy and Wild, 1998). By E19, MC4R mRNA is widely expressed throughout the brain (Mountjoy and Wild, 1998).

## Hormonal Regulation of POMC Neurons during Early Life

Accumulating evidence suggests that there are physiological differences in the regulation of energy balance between adults and neonates. For example, in sharp contrast to the effects of leptin on adults, several groups have reported that exogenous leptin does not significantly inhibit growth, food intake, or energy expenditure until after weaning (Mistry et al., 1999; Ahima and Hileman, 2000; Schmidt et al., 2001; Proulx et al., 2002). Similarly, in contrast to the well-known orexigenic effects of ghrelin in mature animals, exogenous ghrelin does not significantly promote milk intake in the first 2–3 postnatal weeks (Piao et al., 2008; Steculorum and Bouret, 2011a). The general thinking has been that the neonatal brain is relatively insensitive to metabolic hormones. However, both leptin and ghrelin receptors are expressed in the ARH during early postnatal life (Caron et al., 2010; Steculorum and Bouret, 2011a) and these receptors can initiate cellular responses, particularly in POMC neurons. First, acute peripheral leptin treatment in mice on P10 induces phosphorylation of STAT3 and ERK (two major leptin receptor signaling pathways) in 20–35% of ARH POMC neurons (Caron et al., 2010; Bouret et al., 2012). The same leptin treatment increases *Pomc* mRNA levels in the rat ARH (Proulx et al., 2002). Second, acute peripheral ghrelin treatment in P10 mice causes a reduction in *Pomc* gene expression in the ARH (Steculorum and Bouret, 2011a). Collectively, these findings provide convincing evidence that POMC neurons contain functional receptors for metabolic hormones such as leptin and ghrelin during the postnatal period, and suggest that the “hormone insensitivity” observed during this period could instead be from a failure of these cells to relay hormonal signals to other parts of the hypothalamus.

## Mechanisms Underlying POMC Neuron Development

The process of developing highly specialized cellular structures, such as POMC neurons require tight temporal and regional regulation of expression for specific sets of genes and developmental cues. A variety of genetic tools are now available to specifically identify the cellular and molecular pathways that are involved in POMC neuronal development.

### Mechanisms underlying cell fate specification: The role of transcription factors

While the specific programs involved in the determination of POMC terminal fates are unknown, recent data investigating the role of the Mash1-neurogenin 3 (Ngn3) pathway provides some clues as to the role of these transcription factors in the differentiation of the POMC lineage. Using a mouse model of Mash1 deficiency (Mash1−/− mice), McNay et al. (2006) found that this basic helix-loop-helix transcription factor has a pro-neural function and act upstream of Ngn3 to regulate neurogenesis in the ventral hypothalamus. Loss of Mash1 blunts Ngn3 expression in ARH progenitors and is associated with a dramatic reduction in the number of POMC-expressing cells at E12. More recent genetic fate mapping and loss of function studies in mice further demonstrated that the expression of Ngn3 in progenitor cells promotes the development of arcuate POMC neurons (Pelling et al., 2011). Based on the recent observations that a subset of *Pomc*-expressing progenitors in the ARH differentiates into functional mature NPY neurons (Padilla et al., 2010), it would also be interesting to determine the molecular mechanisms that underlie this developmental switch.

### Cues involved in axon growth and axon guidance

The precise molecular mechanisms that are responsible for the formation of POMC circuits are only beginning to be understood. In general, axon development comprises two aspects: the physical act of extension and the molecular mechanisms underlying this process. Axons grow by sending out a highly plastic and sensitive structure called a “growth cone,” which travels toward the target and trails behind it the elongating neurite. As noted above, neonatal POMC neurons express LepRb, and the administration of leptin to mouse neonates results in the activation of major LepRb signaling pathways, including pSTAT3 and pERK in POMC neurons, specifically (Caron et al., 2010; Bouret et al., 2012). Recent anatomical data showed that one of the key factors in controlling the development of POMC neural circuits appears to be the expression of LepRb by POMC neurons (Table 1). Direct exposure of ARH explants *in vitro* to leptin promotes axon growth (Bouret et al., 2004b). In addition, mice or rats that lack functional LepRb signaling (Lepr^db^/Lepr^db^ mice and fa/fa rats, respectively) display a reduced density of ARH POMC projections to the PVH (Bouret and Simerly, 2007; Bouret et al., 2012). Similarly, the density of αMSH-immunoreactive fibers is also markedly reduced in the PVH of s/s mice that lack functional LepRb → STAT3 signaling (Bouret et al., 2012). This observation raises the importance of this signaling pathway, specifically, in the development of POMC neural projections. However, not all LepRb signaling pathways play a role in the formation of POMC projections. For example, mice that lack LepRb → ERK signaling (l/l mice) display comparable densities of αMSH-immunoreactive fibers in the PVH compared to wild-type mice (Bouret et al., 2012). Of particular importance is the fact that leptin appears to exert its developmental action on POMC neural projections during a discrete developmental critical period. POMC neuronal projections are disrupted in leptin-deficient (Lep^ob^/Lep^ob^) mice, and exogenous leptin treatment during the first 2 weeks of postnatal life rescues these projections (Bouret et al., 2004b). In contrast, the treatment of adult Lep^ob^/Lep^ob^ mice with leptin is relatively ineffective and does not increase the density of αMSH fibers in the PVH to levels that are characteristic of wild-type mice (Bouret et al., 2004b).

**Table 1:** **List of genetic and pathological conditions that alter development of POMC-derived neural projections**.

Animal model	Reference
Leptin-deficient (Lep^ob^/Lep^ob^) mouse	Bouret et al. (2004b)
Leptin receptor (Lepr^db^/Lepr^db^) null mouse	Bouret et al. (2012)
LepRb → pSTAT3 (s/s) null mouse	Bouret et al. (2012)
Obese (fa/fa) Zucker rat	Bouret and Simerly (2007)
Obesity-prone DIO rat	Bouret et al. (2008)
Maternal obesity	Kirk et al. (2009)
Maternal diabetes	Steculorum and Bouret (2011b)
Maternal caloric restriction	Delahaye et al. (2008)
Maternal low-protein diet	Coupe et al. (2010)
Postnatal overfeeding	Bouret et al. (2007)

Growing POMC axons must then choose a path to follow and must decide the direction to go on this path to innervate the proper nucleus (e.g., the PVH). The pathways are defined by cell–cell interactions and diffusible chemorepulsive and chemoattractive cues (Tessier-Lavigne and Goodman, 1996). Notably, the diffusible axon guidance cues Netrin, Slit, and Semaphorins are highly expressed in the PVH during development (Xu and Fan, 2008), and POMC terminals express the semaphorin receptor neuropilin 1 (LeGuern and Bouret, unpublished data). Supporting a role for neuropilins/semaphorins in POMC axon guidance, a loss of neuropilin 1 receptors in POMC neurons disrupts the development of POMC axonal projections to the PVH, specifically (LeGuern and Bouret, unpublished data). The formation of POMC neural connections also likely involves cell adhesion molecules. Supporting this idea, mice that are deficient in contactin, a cell adhesion molecule that is involved in the formation of axonal projections, display reduced density of αMSH-immunoreactive fibers in the PVH during postnatal development (Fetissov et al., 2005).

### Cellular mechanisms involved in POMC neuron development: Role of autophagy

The development of POMC neurons also requires massive cytoplasmic remodeling. Autophagy is one of the major cellular degradation processes in which parts of the cytoplasm and intracellular organelles are engulfed within double-membraned vesicles, known as autophagosomes (Klionsky, 2007). An important function of autophagy is to promote cell growth, development, and homeostasis, by maintaining a balance between the synthesis, degradation, and subsequent recycling of cellular components (Levine and Klionsky, 2004; Maiuri et al., 2007; Cecconi and Levine, 2008). Recent morphological data revealed that autophagy is constitutively present in the hypothalamus during important periods of development, including in POMC neuron perikarya and processes such as dendrites (Coupe et al., 2012). Consistent with a functional role for autophagy in POMC neurons, conditional deletion of essential autophagy genes, such as the autophagy-related gene (Atg) 7, results in obesity and impaired glucose homeostasis (Coupe et al., 2012; Kaushik et al., 2012; Quan et al., 2012). These metabolic disturbances are associated with neurodevelopmental abnormalities. Neonates lacking ATG7 in POMC neurons display a reduced density of POMC-containing projections to each of their target nuclei, including the PVH, DMH, and LHA (Coupe et al., 2012). These structural abnormalities persist throughout adult life and appear to be the result of a diminished capacity of POMC neurons for extending axons (Coupe et al., 2012). However, not all of the developmental processes are affected by autophagy deficiency. No changes in POMC cell numbers were reported in mice that lacked autophagy in POMC neurons (Coupe et al., 2012; Kaushik et al., 2012; Quan et al., 2012), which suggests that autophagy does not influence neurogenesis or programed cell death and that instead it has a specific role in axon growth.

## Pathological Conditions that Alter Melanocortin System Development

### Maternal obesity and/or diabetes

In the United States, epidemiological studies have estimated that 20% of women are obese when they conceive (Johnson et al., 2006). This disturbing observation highlights the importance of evaluating the outcomes of maternal obesity in the offspring. Maternal high-fat diet (HFD) feeding during pregnancy is most likely the most widely used approach for studying the consequences of maternal obesity. Notably, offspring born to obese females fed an HFD (45–60% of calories from fat) during gestation only or during both gestation and lactation become progressively overweight, hyperphagic, and glucose intolerant, and they display an increase in adiposity (Chen et al., 2009; Kirk et al., 2009). The model of diet-induced obesity (DIO) developed by Levin et al. (1997) also provides a valuable tool for the study of obesity, in part because Levin’s DIO rats share several features with human obesity, including polygenic inheritance. This animal model is therefore particularly well suited for the study of the relative contribution of genetic versus environmental factors in metabolic programing. Impaired organization of POMC neural circuits is a common feature of maternal obesity (Table 1). Animals born to either high-fat fed or obese-prone DIO dams display a reduced density of αMSH fibers innervating the PVH (Bouret et al., 2008; Kirk et al., 2009). In DIO rats, the abnormal development of POMC projections appears to be the result of a diminished response to the neurotrophic action of leptin during the early postnatal period (Bouret et al., 2008). In addition, a significant remodeling of synapses onto POMC neurons has been observed in DIO rats, particularly in response to nutritional challenges (Horvath et al., 2010). DIO rats fed a chow diet display increased inhibitory inputs to POMC neurons compared to obesity-resistant DR rats. In addition, DIO rats fed a HFD display a loss of synapses onto POMC neurons, whereas high-fat feeding in control (DR) rats causes an increase in POMC synaptic coverage (Horvath et al., 2010).

However, one caveat to keep in mind is that almost all of the animal models of maternal obesity are also hyperglycemic and insulin-resistant. This complication makes it difficult to differentiate the detrimental effects of maternal obesity *per se* as opposed to maternal diabetes. Nevertheless, the manipulation of glucose and insulin levels without an alteration of the diet can be performed experimentally by injecting streptozotocin, a pancreatic beta-cell toxin. Using this approach, we recently found that maternal diabetes alone (i.e., without maternal obesity) can cause a reduction in the density of αMSH-immunoreactive projections to the PVH (Steculorum and Bouret, 2011b) (Table 1). Importantly, an increase in the POMC cell number is observed in the ARH of pups that were born to diabetic dams (Steculorum and Bouret, 2011b), which supports the hypothesis that the low density of POMC-derived fibers is more likely the result of alterations in POMC axon growth as opposed to a reduction in cell numbers.

### Maternal malnutrition

Interest in maternal malnutrition is also particularly high given its high prevalence in underdeveloped countries and the evidence regarding its disease-promoting potential in offspring. Animal models commonly used to study the consequences of maternal malnutrition include caloric restriction and a low-protein diet during pregnancy and lactation. Maternal malnutrition (caused either by caloric restriction or a low-protein diet) affects the overall organization of POMC neural circuits by altering the density of αMSH-containing axons (Delahaye et al., 2008; Coupe et al., 2010) (Table 1). However, if pups born to malnourished dams do not exhibit a rapid catch-up growth, this reduction of POMC innervation is associated with an increased sensitivity to the anorexigenic effect of the melanocortin agonist MTII (Stocker et al., 2012). Therefore, the timing of catch-up growth appears to be an important determinant for the lifelong regulation of the melanocortin system. Supporting this hypothesis, although early catch-up growth ameliorates the abnormal organization of POMC pathways observed in pups born to protein-restricted dams and is highly beneficial for markers of brain development, including markers of cell adhesion and axon elongation, late catch-up growth causes permanent structural defects of POMC neural projections (Coupe et al., 2010).

### Postnatal overfeeding/obesity

Because of the importance of hypothalamic development during postnatal life, including the melanocortin system, in rodents, animal models of postnatal metabolic programing are particularly relevant. An animal model that has proven to be extremely fruitful for the study of postnatal overfeeding is the divergent litter size model. In this model, pups are raised in small litters (SL) from birth to weaning to induce accelerated growth during the pre-weaning period. Postnatally overfed animals show increased adult body weight and an accelerated and exacerbated weight gain when fed an HFD (Glavas et al., 2010). Chronic postnatal overfeeding is associated with a reduced activity of the melanocortin system. For example, rats raised in SL display an overall decrease in the expression of *Pomc* gene expression (Srinivasan et al., 2008; Chen et al., 2009). The observed changes appear to reflect an acquired mechanism that originates from a malprograming of the hypothalamic melanocortin system during early life rather than being a consequence of metabolic dysfunctions, such as overweight and hyperphagia. Consistent with this idea, changes in POMC innervation to the PVH are observed as early as during the first postnatal weeks in neonatally overfed mice, i.e., prior to the development of overweight and hyperphagia (Bouret et al., 2007). In addition to its adverse effects on the *Pomc* gene expression, postnatal overfeeding affects the neuronal response to melanocortins. For example, PVH neurons of chronically overfed pups display reduced electrophysiological responses to αMSH (Davidowa et al., 2003).

## Epigenetic Changes

Epigenetic mechanisms of gene regulation, such as DNA methylation and histone modifications (e.g., methylation, acetylation, ubiquitination) can regulate gene expression in response to environmental stimuli. Given that *Pomc* expression can be affected by the prenatal or early postnatal diet (see above) many groups have investigated whether epigenetic modifications of the POMC promoter occur during the neonatal period in response to nutritional insults. Using a bisulfite sequencing approach, Plagemann and colleagues studied the methylation status of CpG dinucleotides of the *Pomc* promoter in rats that were raised in SL and found that postnatal overfeeding causes hypermethylation of the *Pomc* promoter. Interestingly, this hypermethylation occurs within the two Sp1-related binding sequences of the *Pomc* promoter (Sp1, NF-KB), which are essential for mediating the effect of leptin and insulin on *Pomc* gene expression (Plagemann et al., 2009). Similarly, recent human studies showed that childhood obesity is associated with POMC hypermethylation (Kuehnen et al., 2012). In contrast, methylation of the fetal hypothalamic *Pomc* promoter is reduced in underfed sheep, which is associated with reduced DNA methyltransferase activity and altered histone methylation and acetylation (Begum et al., 2012). In addition, rat neonates from protein-restricted dams display a reduction of hypothalamic Dnmt1 and Dmnt3a mRNA expression (Coupe et al., 2010).

## Conclusion

At a time when obesity, including in children, is reaching epidemic proportions, it appears crucial to better understand the biological processes that mediate the development of metabolic systems. The melanocortin system plays an important role in this process, and recent studies are providing knowledge on how POMC- and MC4R-containing neurons develop. These studies have also revealed that changes in the hormonal and nutritional milieu during critical periods of life can permanently influence the development and functional activity of the melanocortin system. This finding could represent a key mechanism for effecting long-term changes in weight regulation in response to perinatal insults. It also opens new avenues for understanding congenital eating disorders, such as Prader–Willi syndrome. However, if we want to design therapeutic interventions to reverse the metabolic programing of the fetus and/or neonate, it remains to show the periods of vulnerability for the melanocortin system in humans, which most likely proceeds on a timeline of months, compared to days in rodents.

## Conflict of Interest Statement

The authors declare that the research was conducted in the absence of any commercial or financial relationships that could be construed as a potential conflict of interest.
